# The Great Impostor: Transaminitis Masking the Coinfection of Syphilis and Human Immunodeficiency Virus

**DOI:** 10.1155/2017/2481961

**Published:** 2017-03-19

**Authors:** Sunit Tolia, Hassan Kassem, Ana Capatina-Rata

**Affiliations:** St. John Providence Southfield Hospital, Southfield, MI, USA

## Abstract

*Introduction*. The incidence of syphilis continues to rise in the United States over the past 15 years. This disease process is classified into stages and may present with a coinfection of Human Immunodeficiency Virus (HIV).* Case Report*. We present a case of a 32-year-old African American male who presented with cutaneous manifestations of secondary syphilis and transaminitis. A workup revealed that the transaminitis was secondary to underlying syphilitic hepatitis in the presence of HIV coinfection. The patient had a reactive rapid plasma reagin (RPR) of 1 : 64 TU and reactive* Treponema pallidum* particle agglutination assay (TPPA). Lab findings showed alkaline phosphate (ALP) of 648 unit/L, aspartate aminotransferase (AST) of 251 unit/L, and alanine aminotransferase (ALT) of 409 unit/L.* Conclusion*. Syphilitic hepatitis is a recognized entity in the medical literature. It is a manifestation of secondary syphilis and it is more commonly seen in coinfected patients with both syphilis and HIV. Therefore, primary care physicians should keep infectious etiologies (e.g., syphilis and HIV) in the differential diagnosis of patients who present with unexplained liver dysfunction in a cholestatic pattern.

## 1. Introduction

Syphilis has been present prior to the preantibiotic era and still remains to live up to Sir William Osler's description as the “great imitator” in medicine [1]. Syphilis is a sexually transmitted disease caused by* Treponema pallidum* and it can manifest as primary, secondary, early or late latent, and tertiary disease.

Since 2000, the number of syphilitic infections continues to rise in the United States. According to the Center for Disease Control and Prevention (CDC), the total number of reported cases of all stages of syphilis in the USA during the year 2014 was 63,450, a mere 12.3% increase from the previous year of 56,482 cases making it the highest recorded number of cases since 1995 [2]. The coinfection with syphilis and HIV is common because the patient population usually had similar risk factors such as men who have sex with men (MSM), use of recreational drugs, and unprotected sexual practices [3].

## 2. Case Report

A 32-year-old heterosexual African American male presented with a chief complaint of “abdominal pain.” The pain started about a month ago and it was getting progressively worse. It was constant, 8 out of 10 in intensity and throbbing sensation involving the right upper and lower quadrants. It was present throughout the day and worse at night with no improving or worsening factors. Associated symptoms included fever, chills, night sweats, nausea, and generalized weakness.

He did not have any significant past medical or surgical histories. He was unemployed and denied any use of recreational drugs. He was sexually involved with one female partner but did not engage in safe-sex practices.

**Figure 1:**
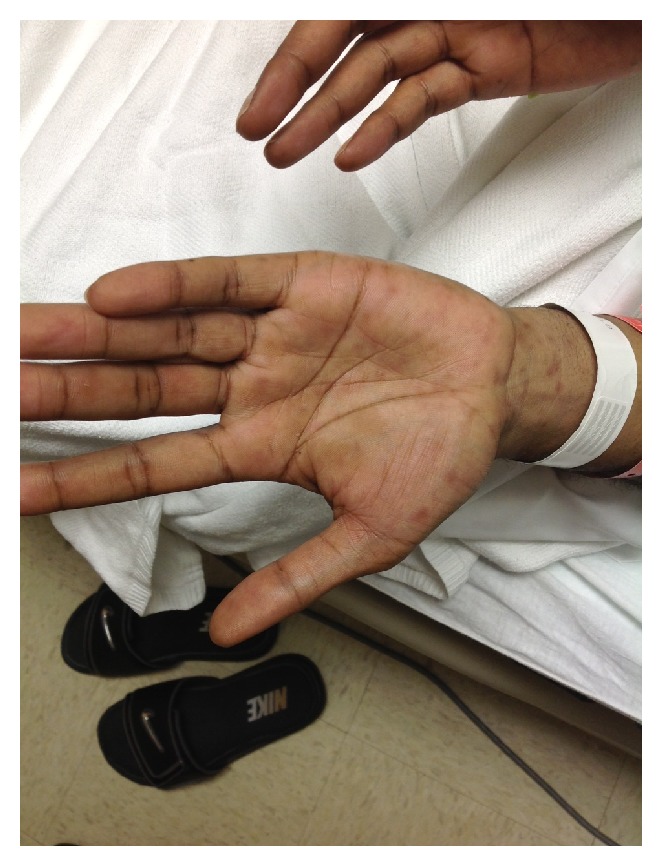
Photograph demonstrating a maculopapular rash distributed on the palms of hand bilaterally.

**Figure 2:**
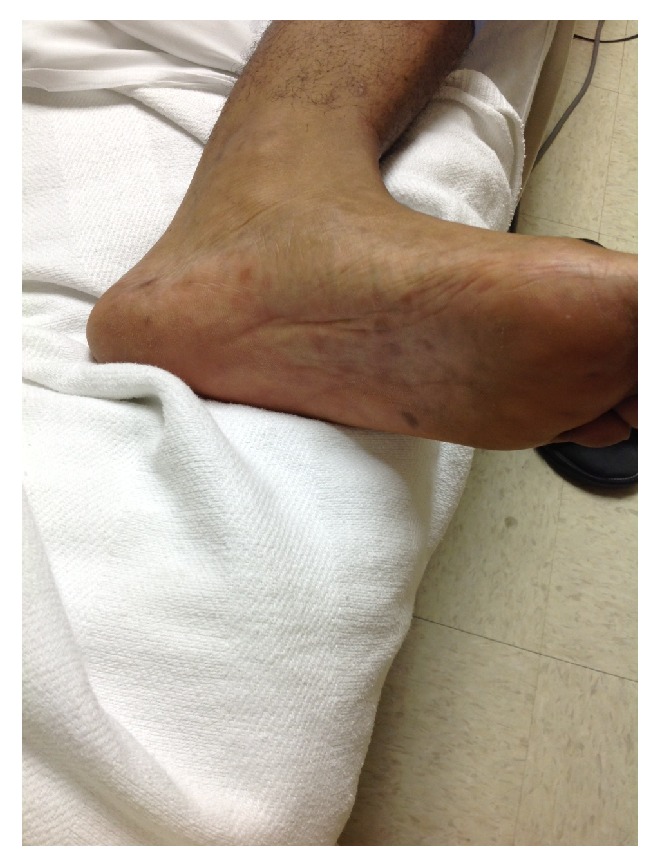
Photograph demonstrating a maculopapular rash distributed on the sole of the feet bilaterally.

**Figure 3:**
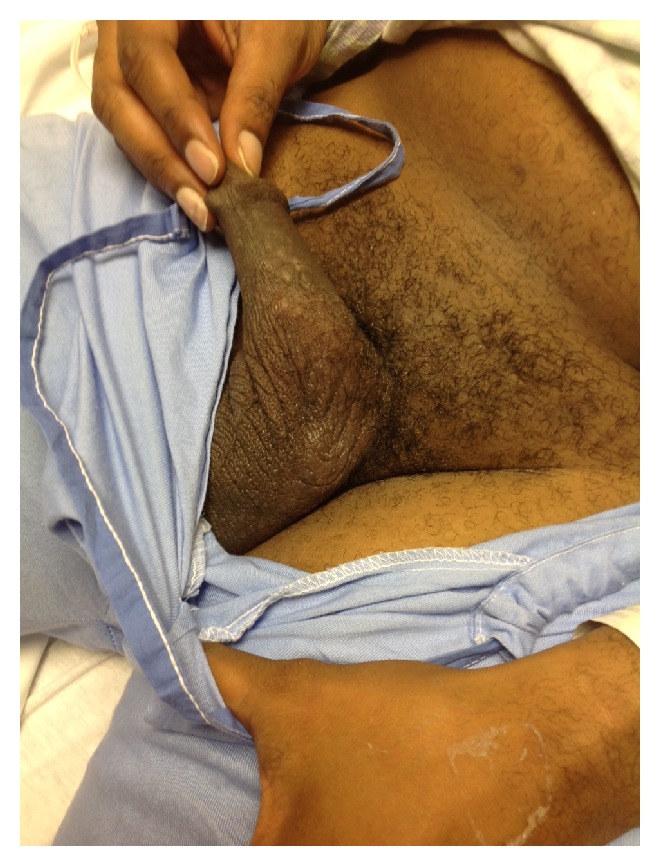
Photograph demonstrating a maculopapular rash distributed on the shaft of penis.

On physical examination, a small 1-2-millimeter, nonraised maculopapular skin rash was present bilaterally on the palms of the hands (Figure 1), sole of the feet (Figure 2), and shaft of the penis (Figure 3). The maculopapular rash was nonerythematosus, nonblanching, and not tender to touch. He had abdominal tenderness to deep palpation in the right upper and lower quadrants with mild scleral icterus and pallor conjunctiva.

Admitting labs were concerning for a cholestasis etiology with total bilirubin at 2.5 mg/dL, ALP of 648 unit/L, AST of 251 unit/L, and ALT of 409 unit/L. The abdominal ultrasound and magnetic resonance cholangiopancreatography were unremarkable. An infectious and autoimmune workup was initiated (Table 1) because of the worsening transaminitis.

In the clinical setting of secondary syphilis, the patient had a reactive RPR titer of 1 : 64 TU and reactive TPPA. He was treated with two doses of Penicillin G benzathine intramuscularly and he subsequently experienced a self-limiting Jarisch–Herxheimer reaction. His transaminitis and ALP marginally improved without complete resolution. Furthermore, his HIV screen was positive with a HIV-1 viral load of 115,000 copies/mL and CD4^+^ cell count of 598 mm^3^. He was started on antiretroviral treatment and a lumbar puncture (LP) was performed. CSF analysis showed a cell count of 4/mm3 (100% lymphocytes), reactive venereal disease research laboratory (VDRL) titer level of 2 TU, protein of 21.7 mg/dL, and glucose of 68 mg/dL (Table 2).

Based on the CSF findings, the patient did not meet the criteria for neurosyphilis. However, due to the persistent headaches, infectious disease team recommended treatment with intravenous Penicillin G potassium 14 million units. At follow-up, the transaminitis and ALP trended down and after completion of the antibiotics his liver dysfunction had resolved and RPR titers were negative.

## 3. Discussion

Primary syphilis presents as painless chancre about 2 centimeters in size in the genital area, usually two to three weeks after sexual activity and it is associated with local lymphadenopathy. According to the literature, of those who are coinfected with syphilis and HIV, greater than 25% present with multiple chancres [4]. However, our patient did not have manifestations of primary syphilis. But it is important to realize that due to the painless nature of primary lesions, they are often under recognized and of less concern to the patients.

Primary syphilis, if left untreated, progresses to secondary syphilis in six to eight weeks via a hematogenous spread. It presents as a maculopapular rash involving the face and extremities, mucosal lesions, and condylomata lata. Other manifestations include arthralgia, sore throat, and fever. Similarly, our patient also had constitutional symptoms along with cutaneous and laboratory findings consistent with secondary syphilis.

The different phases of syphilis are well defined; however, the presentation can differ if the patient is coinfected with other infections. Initially the patient was thought to have a cholestasis etiology; however, this was ruled out based on the diagnostic tests performed. The working diagnosis became syphilitic hepatitis in the setting of vague constitutional symptoms, transaminitis, and positive RPR and TPPA.

Syphilitic hepatitis was first recognized in 1585 and was known as “Luetic Jaundice” [5]. This was commonly seen in patients who are coinfected with both syphilis and HIV. Mullick et al. showed that higher treponemal levels correlate to higher RPR titers and patients are at a greater risk of organ involvement [6]. More specifically, studies have described that those who are coinfected are prone to having liver dysfunction. A multicenter study in Malaga, Spain, looked at 147 cases (January 2004–December 2010), in which 48 cases (32.7%) had coinfection with syphilis and HIV. Liver dysfunction was seen in 45 cases (30%) and the only associated factor is a RPR titer > 1 : 64 [7]. Crum-Cianflone et al. also illustrated that 38% (12/32 cases) of those patients that were HIV positive showed liver enzyme abnormalities [8].

Syphilitic hepatitis can be diagnosed based on abnormal liver function test or a liver biopsy. Laboratory parameters include liver enzymes greater than or equal to 1.25 times the upper limit of normal within a 90-day period prior to the diagnosis, the absence of an alternative cause of hepatitis, and resolution of abnormal labs after the treatment of syphilis [9]. Studies show that there is disproportionate elevation in ALP as compared to AST and ALT. Our patient had similar lab abnormalities, which resolved after being treated with antibiotics. In contrast, liver biopsy is another diagnostic modality, but it is underutilized due to its invasiveness and cost. We did not perform a liver biopsy; however, histologically syphilitic hepatitis causes infiltration of inflammatory cells in portal area, which cause intralobular bile duct degeneration leading to mild hepatitis and periportal hepatocyte necrosis [10]. Jung et al. conducted a retrospective study, in which 100 out of 1,599 HIV patients were coinfected with syphilis [11]. However, only 19 of them had liver involvement and 6 of the 19 were asymptomatic. According to them, histological testing is the gold standard for diagnosing syphilitic hepatitis. However, because the liver dysfunction is rapidly corrected after appropriate treatment, liver biopsy is rarely done.

After the patient was diagnosed with syphilis, we also screened him for other sexually transmitted diseases (STDs). According to the 2015 CDC STD Treatment Guidelines, HIV screening is recommended for all persons who seek evaluation or treatment for STDs and testing should be performed at the time of STD diagnosis in populations that are at high risk of HIV infection [12]. The patient's HIV screen was positive and the results were confirmed once his HIV-1 viral load was noted to be 115,000 copies/mL and CD4^+^ cell count was 598 mm^3^. Infectious disease was consulted and he was started on antiretroviral therapy and Penicillin G benzathine intramuscularly to treat the secondary syphilis.

Tertiary syphilis is a rare condition in the postantibiotic era because of proper preventive measures taken to stop progression. It can present as neurosyphilis, gummatous syphilis, or aortitis. In the preantibiotic era, 30% of syphilis cases progressed to asymptomatic or symptomatic neurosyphilis compared to less than 3% in the postantibiotic era [13]. Neurosyphilis can occur at any stage and is seen at a higher rate in patients coinfected with HIV [14, 15].

There are no current guidelines for obtaining a lumbar puncture in patients with HIV presenting with high RPR titers in the setting of primary or secondary syphilis. In 2006, the CDC STD Treatment Guidelines stated that a serum RPR of 1 : 32 seems to be the best cutoff point to perform an LP because its sensitivity is 100% and specificity is 40% [16]. However, recently CDC has changed their recommendation regarding the need of LP in this patient population. Most recent 2015 guidelines advocate the need for LP if patients have neurological, ophthalmological, or audiological findings in the presence of syphilitic infection. In addition, they state that there are some “soft” indications such as self-reported symptoms (e.g., headaches, blurry vision, and hearing loss), which may not be easily validated on physical exam. Some experts agree that neurosyphilis in HIV-positive patient can be detected more consistently if the CD4 count is less than or equal to 350 cells/ml and RPR or VDRL is greater than or equal to 1 : 32 [17]. Based on the clinical findings, our patient underwent a lumbar puncture because he had several days of bilateral frontal headaches without neurological deficits and serum RPR titers greater than 1 : 32, as well as unknown duration of syphilis infection.

Our patient's CSF results showed a WBC of 4 cells/mm3 (100% lymphocytes), protein of 21.7 mg/dL, glucose of 68 mg/dL, and reactive VDRL with titers of 2 TU. According to Marra et al., CSF-VDRL reactivity remains the essential tool for diagnosing neurosyphilis [18]. However, the sensitivity of CSF-VDRL is significantly decreased in immunosuppressed patients (e.g., HIV population); therefore, other diagnostic methods such as CSF cellularity and protein levels are needed to diagnose neurosyphilis. High levels of cellularity and protein are indicative of underlying neurosyphilis in the absence of other diseases. According to Marra et al., HIV-negative patients with pleocytosis of more than 10 cells and CSF protein levels of more than 0.45 mg/dL are indicative of neurosyphilis, whereas those who are coinfected with HIV should have a pleocytosis of 20 cells or more and CSF protein levels more than 0.45 mg/dL [19].

Based on the laboratory findings, our patient did not meet the criteria for neurosyphilis. But due to infectious disease recommendation in the setting of continuous headache and questionable patient compliance at follow-up appointments, he was treated with intravenous Penicillin G potassium 14 million units. Successful treatment of neurosyphilis is noted by resolution of symptoms and VDRL titers declined at least fourfold [18]. Similarly in our case the patient had negative VDRL titers and normalization of RPR titer after treatment.

## 4. Conclusion

Transaminitis is a common laboratory abnormality that is encountered by primary care physicians. However, it needs to be reemphasized that a cholestatic pattern may be the first indication that the patient may have secondary syphilis. Syphilitic hepatitis was first described over 400 years ago, but it is rarely considered in the differential diagnosis of unexplained liver dysfunction. Internists should keep infectious etiologies such as syphilis and HIV in the differential diagnosis of transaminitis because syphilis continues to present as the “great impostor” in medicine.

## Figures and Tables

**Table 1:** 

Lab test	Results
Hepatitis A IgM	Negative
Hepatitis A IgG	Negative
Hepatitis B surface antigen	Negative
Hepatitis B core antibody IgM	Negative
Hepatitis C IgM	Negative
Hepatitis C IgG	Negative
HSV PCR Type 1	Negative
HSV PCR Type 2	Negative
HIV Ab 1 and 2	Reactive
HIV-1 discriminatory assay	Positive
HIV-2 discriminatory assay	Negative
HIV-1 viral load	115,000 copies/mL
HIV-2 viral load	Negative
CD4 cell count	598 mm^3^
ANA	Negative
Antimitochondrial antibody	Negative
Anti-smooth muscle antibody	Negative
GGT level	Negative
Creatinine kinase	Negative
Aldolase	Negative
Serum RPR titer	1 : 64 TU
Treponema pallidum particle agglutination assay (TPPA)	Reactive
Chlamydia DNA probe	Nondetected
*N. gonorrhoeae* DNA probe	Nondetected
Crypto-Ag serum	Nonreactive
Toxo-Ab IgG	<3
Toxo-Ab IgM	0.04

**Table 2:** 

Cerebrospinal fluid (CSF) lab test	Results
Color	Colorless
Fluid appearance	Clear
Xanthochromia	None
VDRL	Reactive
VDRL titers	2 TU
LDH	11 units/L
Protein	21.7 mg/dL
Glucose	68 mg/dL
Lactic acid	1.4 mmol/L
RBC BF	0
WBC BF	4 cells/mm3
PMN BF	100%
Monocyte BF	0%
Eosinophil BF	0%
Basophil BF	0%
Toxo-Ab IgG	<3.0
Toxo-Ab IgM	<3.0
CMV Ab IgG	<0.20
VZV Ab IgG	<10.0
VZV Ab IgM	0.80
